# Effective Interaction between Homo- and Heteropolymer Block of Poly(*n*-butyl acrylate)-*b*-poly(methyl methacrylate-*r*-styrene) Diblock Copolymers

**DOI:** 10.3390/polym15132915

**Published:** 2023-06-30

**Authors:** Sang-In Lee, Min-Guk Seo, June Huh, Hyun-jong Paik

**Affiliations:** 1 Department of Polymer Science and Engineering, Pusan National University, Busan 46241, Republic of Korea; siclub@lxmma.com (S.-I.L.); smk4505@gmail.com (M.-G.S.); 2 LX MMA R&D Center, 188, Munji-ro, Yuseong-gu, Daejeon 34122, Republic of Korea; 3 Department of Chemical and Biological Engineering, Korea University, Seoul 02841, Republic of Korea

**Keywords:** acrylic block copolymer, Flory–Huggins interaction parameter, thermoplastic elastomer, small-angle X-ray scattering

## Abstract

We investigated the segregation behavior of a molten diblock copolymer, poly(*n*-butyl acrylate)-*b*-poly(methyl methacrylate-*r*-styrene) (PBA-*b*-P(MMA-*r*-S)), wherein styrene (S) is incorporated as a comonomer in the second block to modulate the effective interaction between homopolymer and a random copolymer block. The temperature dependence of the effective interaction parameter $\chi_{eff}$ between *n*-butyl acrylate (BA) and the average monomer of the MMA-*r*-S random block was evaluated from small-angle X-ray scattering (SAXS) analysis using the random phase approximation (RPA) approach. The calculated $\chi_{eff}$, as a function of the styrene fraction in the random copolymer block, shows a good agreement with the mean-field binary interaction model. This consistency indicates that the effective interaction between component BA and the average monomer of the random copolymer block is smaller than the interactions between pure components ($\chi_{BA,MMA},\;\chi_{BA,S}$). The present study suggests that the introduction of a random copolymer block to a block copolymer can effectively reduce the degree of incompatibility of the block copolymer system without altering the constituent species, which may serve as a viable methodology in designing novel thermoplastic elastomers based on triblock or multiblock copolymers.

## 1. Introduction

Triblock copolymers, synthesized in a well-controlled manner using techniques such as ligated anion polymerization (LAP) and controlled radical polymerization (CRP), have been successfully commercialized and applied to thermoplastic elastomers (TPEs). These materials are utilized in a wide range of applications, spanning from the automotive industry to house furnishing, as well as in the building industry. In particular, the continued development of LAP and CRP technologies has enabled the synthesis of novel TPEs that can replace the traditional diene-based TPE such as styrene-butadiene rubber or styrene-isoprene rubber [1,2,3], which have limitations associated with poor oxidation stability, thus having poor weatherability due to the presence of unsaturated bonds in the middle diene-based block [4,5,6,7,8,9].

Fully acrylic block copolymers [10,11,12,13,14,15], which are a class of block copolymers, comprising a rubbery poly(alkyl acrylate) block and glassy poly(alkyl methacrylate) block, are emerging as a potential alternative for TPEs due to their good material properties, including optical clarity and robust resistance to oxidation. These unique properties make them particularly suitable for applications such as replacing or enhancing glass material with high impact resistance in the automotive industry, as well as for outdoor applications, specifically for protective coatings and adhesives [16,17,18,19,20,21]. Previous studies have conducted extensive research on the synthesis of block copolymers comprising *n*-butyl acrylate (BA) block and methyl methacrylate (MMA) blocks with various chains architectures [10,11,12,13,14,15,22,23,24]. Some of these studies have also reported their methods for generating elastomers, in addition to their thermomechanical properties.

In the process of designing a triblock copolymer or other block copolymer architectures for use as TPE, the interaction between constituent blocks becomes a critical consideration. This interaction is typically characterized by the Flory–Huggins interaction parameter χ, which serves as a fundamental design variable because it determines decisively the conformational and morphological states of block copolymers, which, in turn, significantly impact the mechanical performance of TPE. For instance, a theoretical report suggests that the content of bridge conformations between nano-domains in the triblock copolymer morphology, which favorably contributes to the mechanical resilience of TPE, scales as $\chi^{-1/9}$ [25], which implies that controlling χ to have less temperature-dependence is one of key factors when developing TPE with resistance to high temperature. Previously, we performed a scattering analysis on the poly(*n*-butyl acrylate)-*b*-poly(methyl methacrylate) (PBA-*b*-PMMA) to measure χ for this fully acrylic block copolymer. This analysis revealed that χ between BA and MMA exhibited a relatively less susceptibility to temperature variation [26] as compared to those in other traditional TPE systems [27,28,29]. This suggests that using BA and MMA block components as the primary constituents for constructing block copolymers holds significant promise. The advantageous properties of these components can be capitalized to develop block copolymers with a variety of chain architectures, including triblock, star, graft, and multiblock configurations, all of which could prove suitable for TPE applications. In these structures, the capability to fine-tune the interaction between the constituent blocks is especially advantageous. This adaptability allows for the precise manipulation of the block copolymer’s properties and behavior, enabling the development of customized materials for specific applications. By adjusting the interaction parameter, we can control the polymer’s morthology and conformational states, thereby influencing its mechanical performance and functionality.

This work demonstrates the control of the effective interaction for the BA-MMA-based block copolymer by replacing the MMA block with a random copolymer block, termed as MMA-r-S, where styrene monomers (S) are randomly incorporated into the MMA block. The MMA and S components have been widely employed as a comonomer pair in the construction of random copolymers, primarily owing to their similar physical properties, such as glass transition temperature and surface tension, which offer an option to control the effective interaction between constituent blocks, while minimally altering the material properties. For TPE applications in the PBA-PMMA system, where the glassy PMMA domain is embedded in the rubbery PBA matrix, we opted to design a random copolymer block P(MMA-*r*-S) rather than P(BA-*r*-S), given the glassy nature of polystyrene. To quantify the effective interaction parameter between BA and MMA-r-S as a function of the fraction of S comonomer (φ), small angle X-ray scattering (SAXS) measurements were performed on molten poly(*n*-butyl acrylate)-*block*-poly(methyl methacrylate-*ran*-styrene) (PBA-*b*-P(MMA-*r*-S)). To accomplish this, a series of PBA-*b*-P(MMA-*r*-S) diblock copolymers were synthesized with varying styrene comonomer fractions via atom transfer radical polymerization (ATRP).

## 2. Materials and Methods

### 2.1. Materials

Butyl acrylate (BA, 99%), methyl methacrylate (MMA, 99.5%), and styrene (99.5%) were purchased from TCI (Tokyo, Japan), Daejung Chemicals & Metals (Siheung, Republic of Korea), and Junsei (Tokyo Japan), respectively. Residual inhibitors in BA, MMA, and styrene were removed by passing them through an alumina column. Copper(I) bromide (CuBr, 98.0%), copper(I) chloride (CuCl, 98.0%), copper(II) bromide (CuBr${}_{2}$, 98.0%), copper(II) chloride (CuCl${}_{2}$, 98.0%), and *N*,*N*,$N^{\prime }$,$N^{\prime \prime }$,$N^{\prime \prime }$-pentamethyldiethylenetriamine (PMDETA; 98.0%) were purchased from Sigma-Aldrich (Seoul, Republic of Korea). CuBr and CuCl were purified by stirring them in glacial acetic acid and washing them with ethanol and diethyl ether. Anisole (98%, Daejung Chemicals & Metals (Siheung, Republic of Korea)) and all other chemicals were used as received.

### 2.2. ATRP Synthesis

**Scheme 1:**
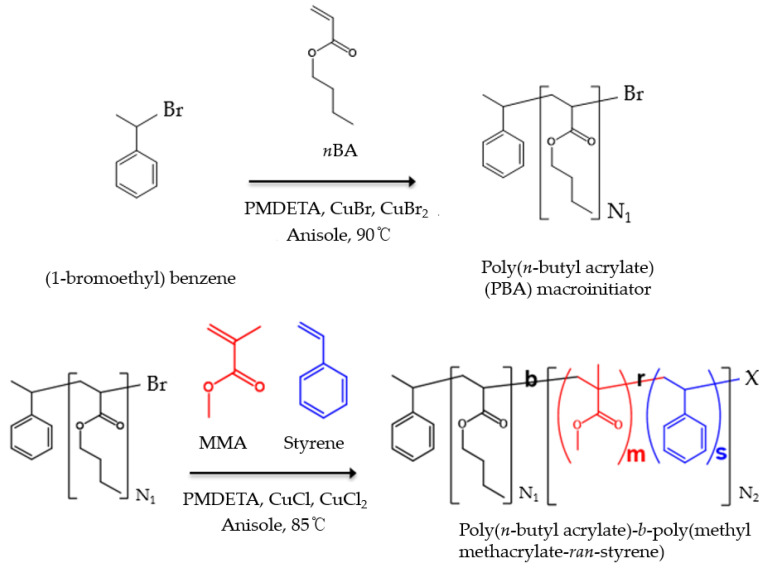
Synthetic scheme of PBA macroinitiator and PBA-*b*-P(MMA-*r*-S) diblock copolymer.

An overall reaction scheme outlining the ATRP synethesis of the PBA macroinitiator and the subsequent formation of the PBA-*b*-P(MMA-*r*-S) diblock copolymer is provided in Scheme 1.

#### 2.2.1. ATRP Synthesis of PBA Macroinitiator

In a 100 mL Schlenk flask, CuBr (152.97 mg, 1.07 mmol) and CuBr${}_{2}$ (4.86 mg, 0.02 mmol) were introduced. Subsequently, butyl acrylate (30.0 mL, 208.27 mmol), (1-bromoethyl) benzene (0.15 mL, 1.09 mmol), PMDETA (0.23 mL, 1.09 mmol), and anisole (20.0 mL) were introduced to the flask using N${}_{2}$ purged syringes. The mixture was then subjected to reaction in an oil bath at 90 °C. Upon reaching 40% monomer conversion (as measured by gas chromatography), the polymerization was halted by exposure to air and the addition of THF. The reaction mixture was subsequently passed through a neutral alumina column to eliminate the Cu catalyst. The resulting polymer was isolated through precipitation into a methanol/water (8:2) solution, followed by filtration and drying under a vacuum. Three PBA macroinitiator polymers were synthesized in total, with molecular weights determined using size-exclusion chromatography (SEC) as follows: $M_{n}$ = 12,500 g/mol and PDI= 1.08; $M_{n}$ = 17,100 g/mol and PDI = 1.09; $M_{n}$ = 28,000 g/mol and PDI = 1.05.

#### 2.2.2. ATRP Synthesis of PBA-*b*-P(MMA-*r*-S) Diblock Copolymer

Into a 50 mL Schlenk flask, CuCl (122.0 mg, 0.91 mmol), CuCl${}_{2}$ (7.50 mg, 0.02 mmol), degassed methyl methacrylate (6.18 mL, 58.0 mmol), styrene (0.14 mL, 1.18 mmol) (comprising 2% of the total monomer), PMDETA (0.19 mL, 0.93 mmol), and PBA macroinitiator (1 g, 0.09 mmol (M${}_{n}$ = 12,500 g/mol)) were charged in anisole (10.0 mL). The polymerization was performed at 85°C and was abruptly terminated by exposure to ambient air followed by the addition of THF. The Cu catalyst was eliminated from the reaction mixture by filtration through a neutral alumina column. The polymer was obtained from precipitation into methanol. PBA-*b*-P(MMA-*r*-S) diblock copolymers were synthesized using two PBA macroinitiators ($M_{n}$ = 12,500 g/mol and PDI= 1.08; $M_{n}$ = 17,100 g/mol). Similarly, PBA-*b*-PMMA and poly(*n*-butyl acrylate)-*b*-polystyrene (PBA-*b*-PS) were synthesized using the aforementioned procedure. The resulting polymers and their respective molecular weights are summarized in Table 1.

#### 2.2.3. Analytical Techniques

**Figure 1:**
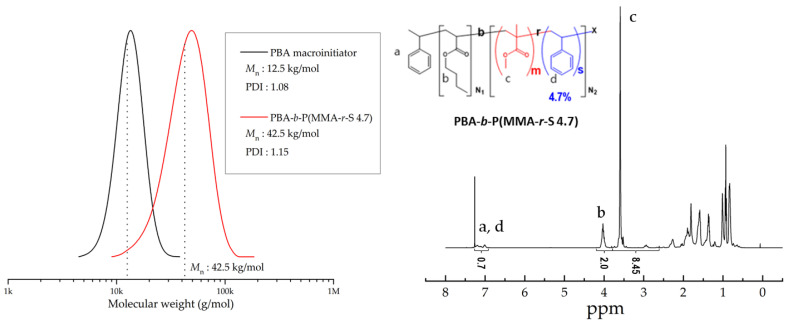
SEC trace and ${}^{1}$H NMR spectrum for PBA-*b*-P(MMA-*r*-S 4.7).

The HP 5890 gas chromatograph, fitted with an HP101 column, was used to verify monomer conversion during each stage of the ATRP process. The number-average molecular weights ($M_{n}$) and polydispersity index (PDI=$M_{w}/M_{n}$) were determined by SEC calibrated with PMMA standards. ${}^{1}$H NMR spectra were obtained using Varian Unity Inova 500 spectrometer and a 500 MHz Agilent Superconducting FT-NMR spectrometer using CDCl${}_{3}$ as solvent. Figure 1 presents the SEC trace and ${}^{1}$H NMR spectrum for PBA-*b*-P(MMA-*r*-S 4.7). The ${}^{1}$H NMR spectrum confirms that the styrene content of the P(MMA-*r*-S) of the sample in Figure 1 is 0.7/5 ÷ (8.45/3 + 0.7/5) = 0.047, where 0.7/5 corresponds to the aromatic protons of S and 8.45/3 corresponds to the methyl protons of MMA. For complete information, the SEC traces and ${}^{1}$H NMR spectra of all synthesized block copolymer samples are provided in Figure 1 and Supplementary Figures S1 and S2.

### 2.3. SAXS Measurements

The synchrotron SAXS experiments were conducted at the 4C SAXS II beamline of the Pohang Light Source II (PLS II), with 3 GeV power, at the Pohang University of Science and Technology (POSTECH), Korea. Detailed information about the optical layout and specifications of the 4C SAXS beamline (BL) can be found in reference [30]. The main optical components of the 4C SAXS II beamline include an in-vacuum undulator, a Si(111) double crystal monochromator, and a vertical focusing toroidal mirror. The experimental conditions were configured with a beam size of 0.15 (V) × 0.24 (H) mm${}^{2}$ and a sample thickness of 1.0 mm. A 2D charge coupled detector (Rayonix 2D SX165, Evanston, IL, USA) was used, and the sample-to-detector distance was positioned at 3.0m, which allows a scattering vector range of 0.01 Å${}^{-1}$ < *q* < 0.2 Å${}^{-1}$. The magnitude of the scattering vector was determined by q=(4π/λ)sinθ, where 2θ is the scattering angle, and the wavelength (λ) of the X-ray beam was 0.734 Å. The scattering angle was calibrated with a polystyrene-*b*-polyethylene-*b*-polybutadiene-*b*-polystyrene block copolymer standard. Background correction was conducted to obtain high-quality SAXS data and to mitigate the effect of polyimide sample-sealed film and air scattering interference, as described elsewhere [31,32,33]. The experimental setup was equipped to maintain accurate temperature control within an accuracy of ± 0.03 °C. Our SAXS measurements were performed in the temperature range of 130–270 °C. This necessitated precise temperature control to prevent potential thermal degradation of the PMMA component in our block copolymer samples [34,35]. Following the SAXS measurement, our samples were examined using GPC and showed no indication of mass loss. The SAXS measurements were carried out in the course of a heating cycle which initiated at 130 °C. Throughout this heating process, the diblock samples were held at a specific temperature for 1h to achieve the greatest possible thermal equilibrium.

### 2.4. Characterization of Diblock Copolymer

**Figure 2:**
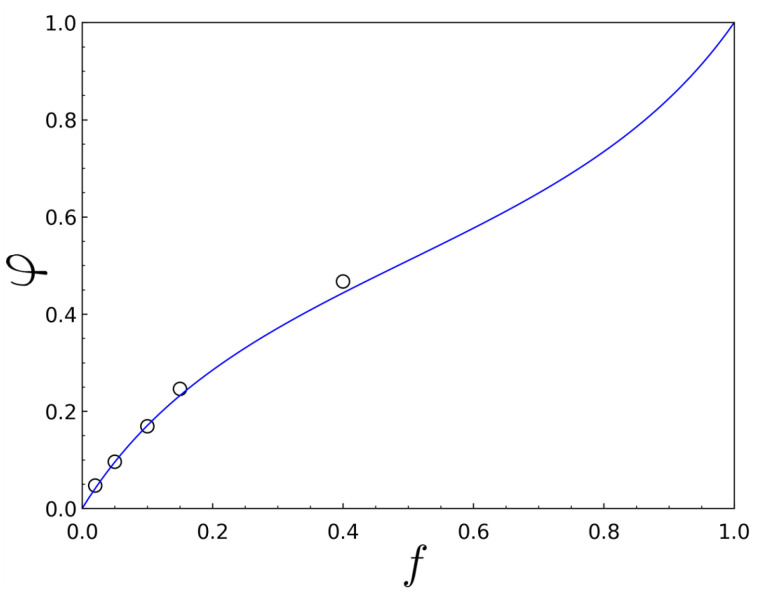
Styrene comonomer fraction (φ) in the second block as a function of the styrene fraction in the feed (*f*). The solid line represents the result using copolymer equation using $r_{1}$ = 0.52 and $r_{2}$ = 0.46.

The PBA-*b*-P(MMA-*r*-S) samples were prepared by ATRP using PBA macro-initiators with $M_{n}$ = 12,500 g/mol (PDI = 1.08) and $M_{n}$ = 17,100 g/mol (PDI = 1.09). The molecular weight during the polymerization increased with time and were in the range of 23.4–43.7 kg/mol in SEC after the quenching of polymerization reactions, whereas the PDI were in the range of 1.13–1.17. The PBA-*b*-PS sample was prepared by ATRP using the PBA macroinitiator with $M_{n}$ = 28,000 g/mol (PDI = 1.05). In SEC, the molecular weight of PBA-*b*-PS polymer was 42 kg/mol and PDI was 1.34 (Table 1). The conversion of methyl methacrylate was estimated to be about 30% on the assumption that the initiation efficiency of the initiator was 100%. The composition of the PBA block was ascertained by comparing the relative areas of the methyl protons (3.75–2.75 ppm) in the PMMA component (-CO${}_{2}$CH${}_{3}$) with those of the methylene protons (4.25–3.85 ppm) in the PBA component (-CO${}_{2}$CH${}_{2}$C${}_{3}$H${}_{7}$), as presented in the ${}^{1}$H NMR spectrum (Figure 1 and Supplementary Figure S2). To estimate the styrene composition in the second P(MMA-*r*-S) block, the relative areas of the methyl protons (3.75–2.75 ppm) in the MMA component (-CO${}_{2}$CH${}_{3}$) were compared to those of the aromatic protons (7.35–6.85 ppm) in the styrene component (-C${}_{6}$H${}_{5}$), as shown in Figure 1 and Supplementary Figure S2c–h. In the case where the second block is comprised solely of MMA, a relatively narrow peak is seen at δ = 3.75–3.25 ppm (-CO${}_{2}$CH${}_{3}$), as shown in Supplementary Figure S2b. However, when the styrene monomer is copolymerized into the P(MMA-*r*-S) block, the methyl protons (-CO${}_{2}$CH${}_{3}$) peaks of the MMA exhibit a broader range at δ = 3.75–2.75 ppm, as shown in Supplementary Figure S2c–h. This peak broadening is attributed to the ring current effect, caused by the adjacent phenyl groups of the styrene monomer units, which results in a shift towards lower chemical shifts (δ) [36]. Furthermore, an increase in the styrene composition of the second block is supported by a higher area ratio of the aromatic protons (7.35–6.85 ppm), compared to the area of the methyl protons (3.75–2.75 ppm), as demonstrated in Supplementary Figure S2c–g. Figure 2, which shows the styrene composition (φ) of the second block determined using NMR spectroscopy as a function of the styrene molar feed (*f*), which agrees well with the copolymer equation using the reactivity ratios $r_{1}$ = 0.52 and $r_{2}$=0.46 in the conventional free radical system and atom transfer radical copolymerization [1,37]. Table 1 summarizes the sample codes and characteristics of the diblock copolymer samples used in this study.

## 3. Results

**Figure 3:**
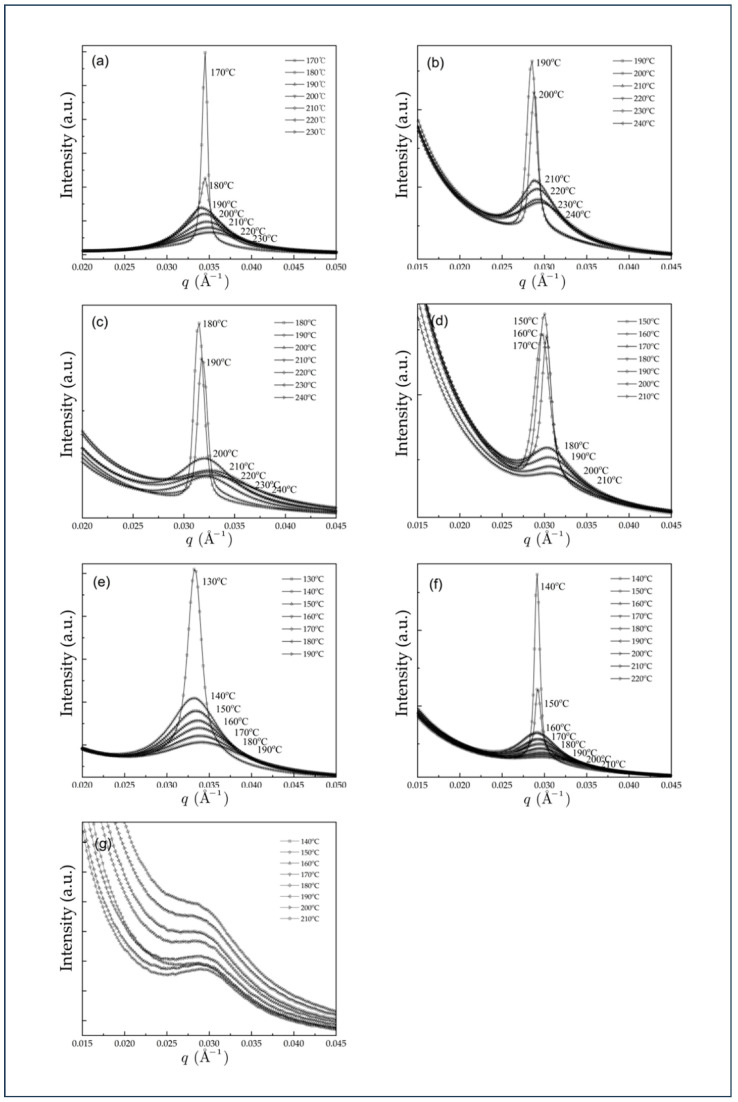
SAXS profiles at various temperatures for block copolymer samples with different φ: (**a**) B${}_{13}$M${}_{11}$; (**b**) B${}_{13}$(M${}_{29}$S${}_{1})$; (**c**) B${}_{13}$(M${}_{17}$S${}_{2})$; (**d**) B${}_{13}$(M${}_{22}$S${}_{5})$; (**e**) B${}_{13}$(M${}_{13}$S${}_{5})$; (**f**) B${}_{17}$(M${}_{15}$S${}_{12})$; and (**g**) B${}_{28}$S${}_{14}$.

Figure 3 shows the SAXS profiles of PBA-*b*-P(MMA-*r*-S) samples obtained at different temperatures (*T*), where the styrene comonomer fraction (φ) in the random block ranges approximately from 0.05 to 0.5, covering the compositions between the diblock samples with homo block samples (i.e., PBA-*b*-PMMA corresponding to φ = 0 and PBA-*b*-PS to φ = 1). These profiles were used for the random phase approximation (RPA) analysis, which quantifies the effective interaction $\chi_{eff}$ between BA and the average monomer of random block, MMA-*r*-S, as a function of *T* and φ. To determine the temperature dependence of $\chi_{eff}$, we used the SAXS profiles of the samples in the disordered temperature regime. For block copolymer samples of B${}_{13}$M${}_{11}$, B${}_{13}$(M${}_{29}$S${}_{1}$), B${}_{13}$(M${}_{22}$S${}_{5}$), B${}_{13}$(M${}_{13}$S${}_{5}$), and B${}_{17}$(M${}_{15}$S${}_{12}$), the SAXS profiles, which were broad in the regime of high temperatures, sharpened, indicating the transition from disorder to an ordered state, whereas the sample B${}_{28}$S${}_{14}$ remained disordered in the investigated temperature regime. After identifying the disordered temperature regimes for each sample, the evaluation of the temperature dependence of $\chi_{eff}$ was carried out using the RPA analysis at a temperature greater than $T_{MF}$, where $T_{MF}$ denotes the crossover temperature that marks the change from a mean-field regime to the non-mean-field regime [38,39,40]. The RPA postulates that in the disordered state, the scattering intensity I(q) at scattering vector *q* is directly proportional to the reciprocal of $\left[\Gamma \left(q,R_{1},R_{2}\right)-2\chi_{eff}\right]$, where Γ represents the second vertex function associated with the single-chain correlation functions in the ideal state and $R_{\alpha }$ denotes the root mean square radius of gyration of the component α (α = 1: BA, α = 2: MMA-*r*-S). The estimation of $\chi_{eff}$ at each temperature was then acquired by fitting $I\left(q\right)/I\left(q^{*}\right)$ using the fitting function $F\left(q;R_{1},R_{2},\chi_{eff}\right)=N_{min}{\left[\Gamma \left(q,R_{1},R_{2}\right)-2\chi_{eff}\right]}^{-1}$ with the fitting parameters, $R_{1}$, $R_{2}$, and $\chi_{eff}$ for a specific set of molecular parameters, where $q^{*}$ is the dominant scattering vector and $N_{min}$ is the minimum value of $\Gamma \left(q,R_{1},R_{2}\right)-2\chi_{eff}$. The comprehensive formulas for the fitting function, which include the molecular parameters, can be found in the Supplementary Materials.

**Figure 4:**
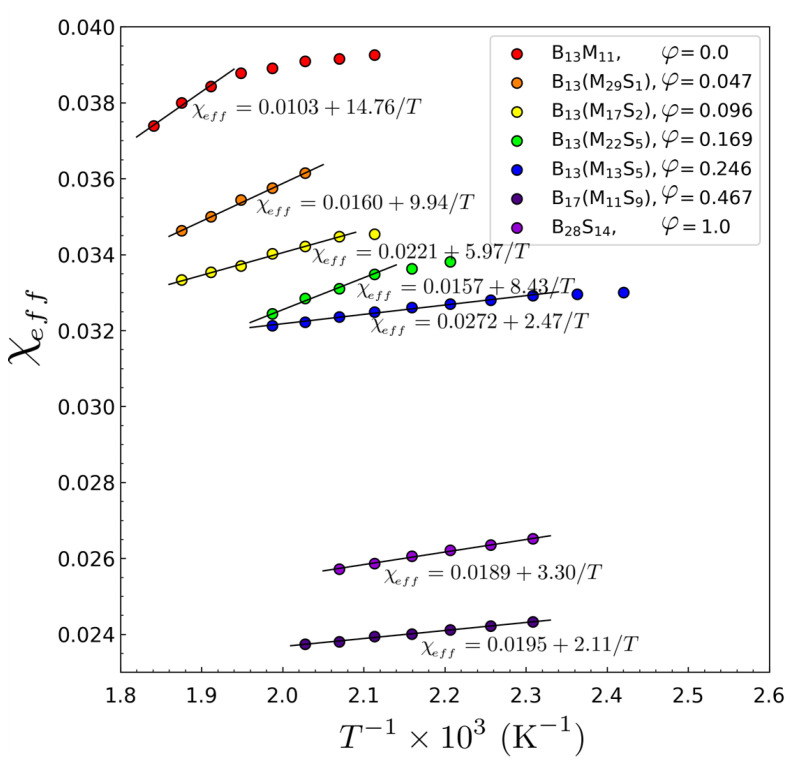
The temperature dependence of $\chi_{eff}$ for block copolymer samples with different φ.

The scattering profiles normalized by $I(q^{*})$ are fitted to $F(q;R_{1},R_{2},\chi_{eff})$ for block copolymer samples at various temperatures in the disordered state, as shown in Supplementary Figure S3. From the fitting curves, the temperature dependence $\chi_{eff}\left(T\right)$ can be obtained using the linear relation $\chi_{eff}=\chi_{S}+\chi_{H}/T$ in the linear regime, where $\chi_{S}$ and $\chi_{H}$ are the entropic and enthalpic contribution to the overall $\chi_{eff}$ [40,41,42,43]. Figure 4 presents the computed $\chi_{eff}$ values plotted against the inverse temperature for various block copolymer samples with different cases of φ, by which the temperature (*T*) and the comonomer effect (φ) on $\chi_{eff}$ can be analyzed. For each case of block copolymer samples in Figure 4, the expression $\chi_{eff}=\chi_{S}+\chi_{H}/T$ in the linear regime is indicated. Figure 4 shows that the interaction parameter between pure components BA and MMA, corresponding to $\chi_{eff}\left(\varphi =0.0\right)$, is larger than that between BA and S to $\chi_{eff}\left(\varphi =1.0\right)$. Figure 4 notably reveals that $\chi_{eff}$ gets a minimum, implicating that the effective interaction between the homo BA block and random MMA-*r*-S block cannot be represented by a linear combination of interaction parameters between homopolymer blocks, i.e., $\chi_{BA,S}\varphi +\chi_{BA,MMA}\left(1-\varphi \right)$, where $\chi_{BA,S}$ and $\chi_{BA,MMA}$ are the interaction parameter between components between BA and S, and between BA and MMA, respectively.

**Figure 5:**
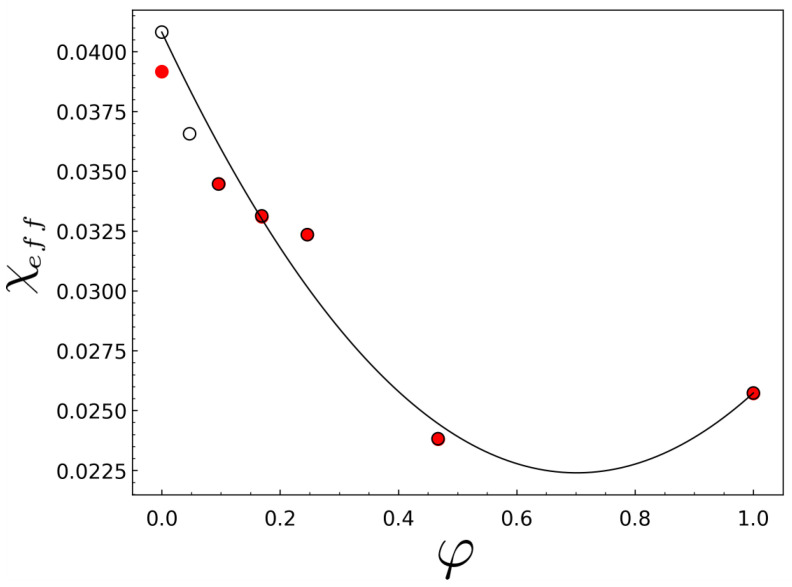
$\chi_{eff}$ for PBA-*b*-P(MMA-*r*-S) as a function of φ at *T* = 200 °C. The filled red circle represents the estimation from fitting using RPA, whereas the open circles represent the estimation based on the formula ($\chi_{eff}=\chi_{S}+\chi_{H}/T$) obtained in the mean-field region. The solid line represents the prediction obtained from the binary interaction model, using the values $\chi_{BA,S}=0.0189+3.30/T$, $\chi_{BA,MMA}=0.0103+14.76/T$, and $\chi_{MMA,S}=0.0282+4.46/T$.

This distinctive minimum behavior exhibited by the comonomer effect $\chi_{eff}\left(\varphi \right)$ can be described within a mean-field approach by the binary interaction model developed from theories on copolymer blends [44,45,46]:

(1) $$\chi_{eff}=\chi_{BA,S}\varphi +\chi_{BA,MMA}\left(1-\varphi \right)-\chi_{MMA,S}\varphi \left(1-\varphi \right)$$

where $\chi_{\alpha ,\beta }$ are the interaction parameter between component α and β (α, β=BA/MMA/S). The first two terms on the right-hand side of Equation (1) represent the contributions due to the contacts between the homo BA block and the random block, and the third term accounts for the contributions due to the contacts between segments belonging to the random block. Figure 5 illustrates a comparison between the experimentally determined $\chi_{eff}\left(\varphi \right)$ and the predictions using Equation (1) at *T* = 200 °C, where the parameters $\chi_{BA,S}=0.0189+3.30/T$, $\chi_{BA,MMA}=0.0103+14.76/T$, and $\chi_{MMA,S}=0.0282+4.46/T$ [43] were used. The strong correlation with theoretical model suggests that the binary interaction model, originally developed for the copolymer blend system, is also effective in describing a block copolymer system incorporating a random copolymer block. Previous studies have similarly employed the binary interaction model to interpret the effective interaction of a block copolymer with a random block [47,48,49]. In those studies, however, the model was applied as a fitting function to extract the segmental interaction parameters $\chi_{\alpha ,\beta }$ between the constituent components (α,β) of the system. In Figure 5, on the other hand, a quadratic relationship, as described by Equation (1), is computed using the independently obtained $\chi_{\alpha ,\beta }$. Conversely, this good agreement validates the use of the binary interaction model, as employed in previous approaches, for estimating $\chi_{\alpha ,\beta }$ in the block copolymer systems having random copolymer blocks.

## 4. Conclusions

To summarize, we have presented an evaluation of the effective interaction parameter between the monomer of a homopolymer block, PBA, and the average monomer of a random copolymer block, P(MMA-*r*-S), in the diblock copolymer PBA-*b*-P(MMA-*r*-S). This was achieved by analyzing SAXS measurements which were adjusted to fit RPA equations across varying temperatures and comonomer compositions in the random block. The estimation of $\chi_{eff}$ reveals that the effective interaction parameter follows a quadratic relationship with respect to the variation in comonomer content, quantitatively in good agreement with the mean-field binary interaction model proposed for the phase behavior of polymer blends involving random copolymers. Our findings further propose that the incorporation of a styrene comonomer into acrylic block copolymers, specifically those based on BA-MMA, offers an option for controlling the effective interaction between constituent blocks, providing greater flexibility in managing the fraction of bridging chains, a factor that is crucial in the development of thermally stable and mechanically resilient TPEs required for weatherproofing materials.

## Figures and Tables

**Table 1:** Characteristics of PBA-*b*-P(MMA-*r*-S x) used in this study. The variable x represents the percentage fraction of styrene in the second block determined using NMR.

Diblock Samples	Code	$M_{n}$ (kg/mol) (PDI) of PBA	$M_{n}$ (kg/mol) (PDI) of Diblock	φ
PBA-*b*-PMMA	B${}_{13}$M${}_{11}$	12.5 (1.08)	23.5 (1.15)	0.0
PBA-*b*-P(MMA-*r*-S 4.7)	B${}_{13}$(M${}_{29}$S${}_{1})$	12.5 (1.08)	42.5 (1.15)	0.047
PBA-*b*-P(MMA-*r*-S 9.6)	B${}_{13}$(M${}_{17}$S${}_{2})$	12.5 (1.08)	31.3 (1.13)	0.096
PBA-*b*-P(MMA-*r*-S 16.9)	B${}_{13}$(M${}_{22}$S${}_{5})$	12.5 (1.08)	39.5 (1.16)	0.169
PBA-*b*-P(MMA-*r*-S 24.6)	B${}_{13}$(M${}_{13}$S${}_{5})$	12.5 (1.08)	30.9 (1.17)	0.246
PBA-*b*-P(MMA-*r*-S 46.7)	B${}_{17}$(M${}_{15}$S${}_{12})$	17.1 (1.09)	43.7 (1.17)	0.467
PBA-*b*-PMMA	B${}_{28}$S${}_{14}$	28.0 (1.05)	42.0 (1.06)	1.0

## Data Availability

The data presented in this study are available on request from the corresponding author.

## Supplementary Materials

The supporting information can be accessed and downloaded at the following link: https://www.mdpi.com/article/10.3390/polym15132915/s1, Figure S1: SEC traces of block copolymer samples, Figure S2: ${}^{1}$H NMR spectra of block copolymer samples, Figure S3: The normalized scattering profiles $I\left(q\right)/I\left(q^{*}\right)$ are fitted to $F(q;R_{1},R_{2},\chi_{eff})$ for block copolymer samples at various temperatures in the disordered state, and Table S1: Molecular parameters of PBA-*b*-P(MMA-*r*-S) used for fitting with the fitting function.
