# Supplementary figures and images for: Effective Interaction between Homo- and Heteropolymer Block of Poly(n-butyl acrylate)-b-poly(methyl methacrylate-r-styrene) Diblock Copolymers
Source: Polymers (Basel). 2023 Jun 30;15(13):2915. doi: 10.3390/polym15132915 (PMC10346336; doi:10.3390/polym15132915)

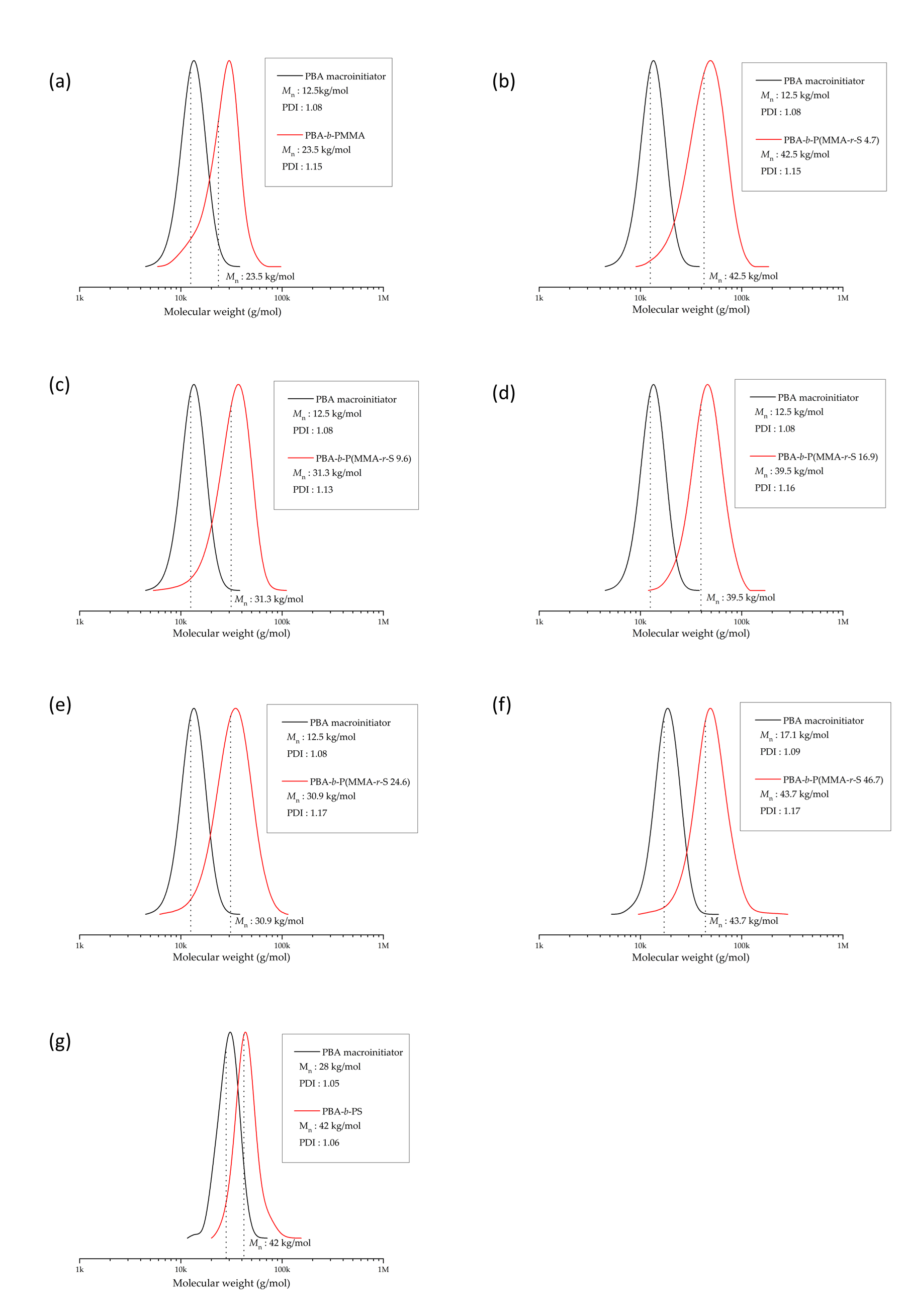

Supplement: Supplementary file 1 [file polymers-15-02915-s001.zip › figS1.png]

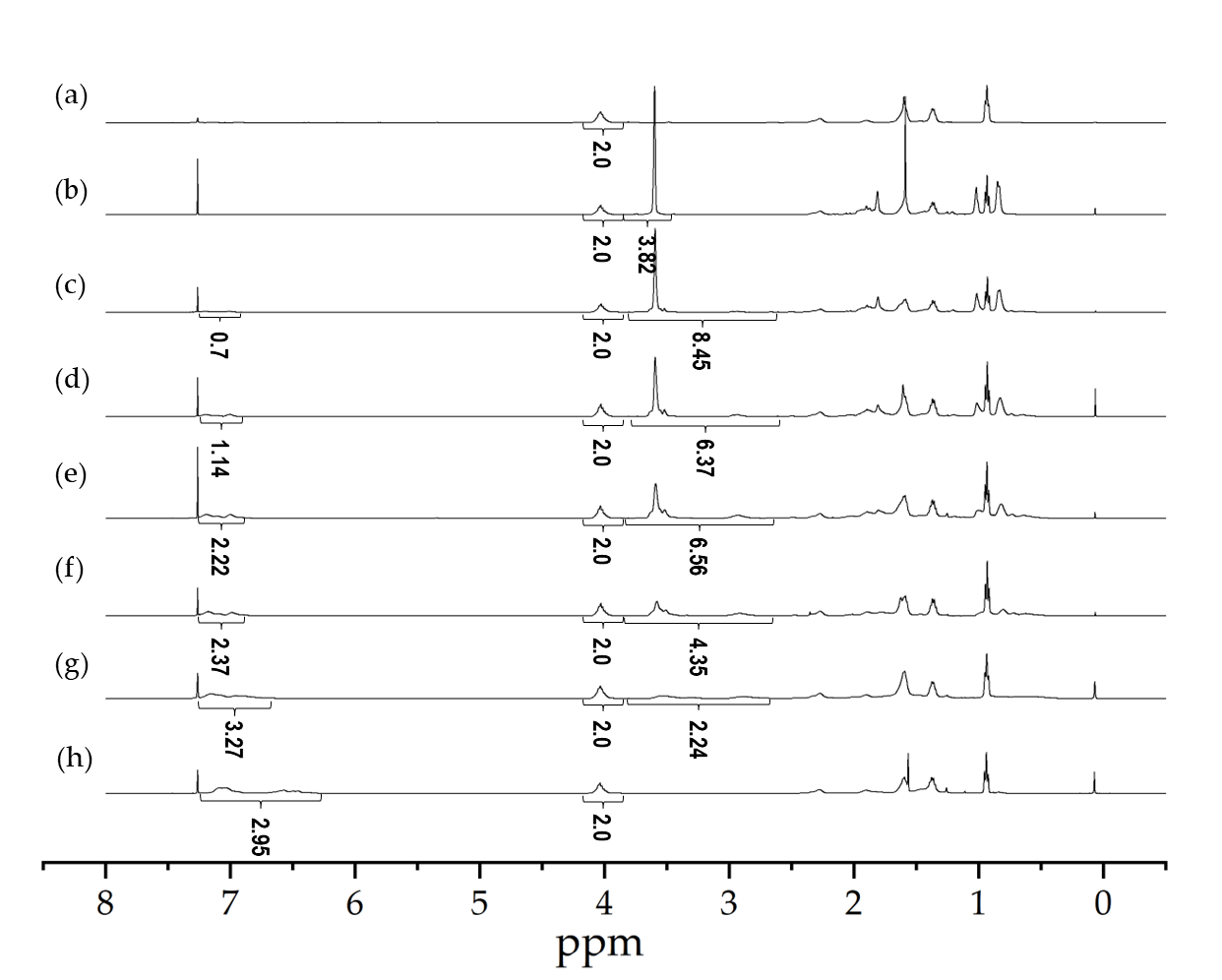

Supplement: Supplementary file 1 [file polymers-15-02915-s001.zip › figS2.png]

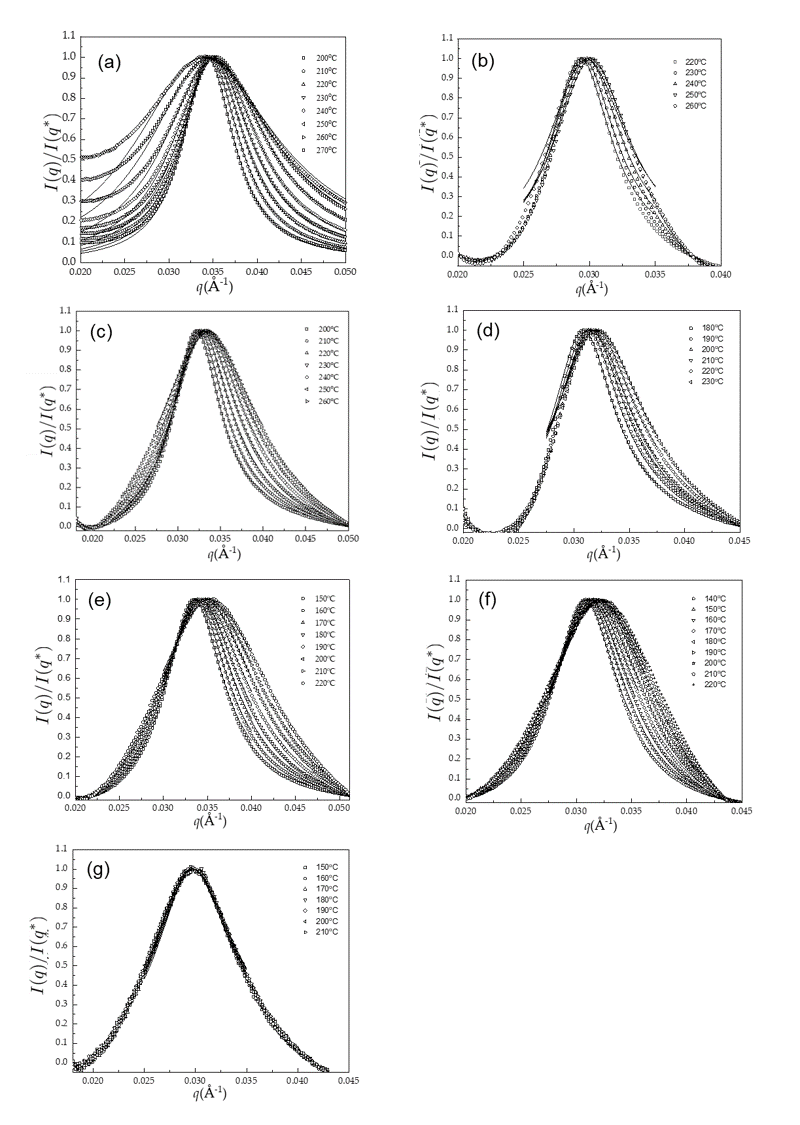

Supplement: Supplementary file 1 [file polymers-15-02915-s001.zip › figS3.png]

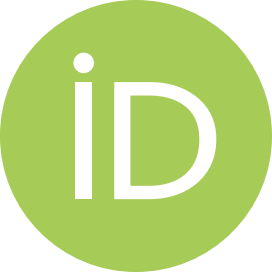

Supplement: Supplementary file 1 [file polymers-15-02915-s001.zip › Definitions/logo-orcid.pdf]

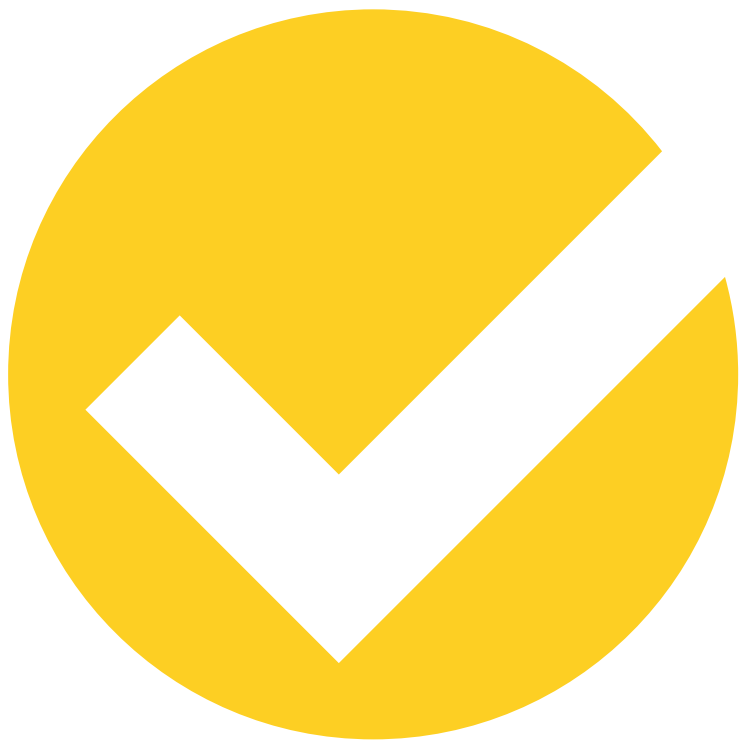

check for  
updates

Supplement: Supplementary file 1 [file polymers-15-02915-s001.zip › Definitions/logo-updates.pdf]
